# Methodology for Whole-Genome Sequencing of Methicillin-Resistant *Staphylococcus aureus* Isolates in a Routine Hospital Microbiology Laboratory

**DOI:** 10.1128/JCM.00180-19

**Published:** 2019-05-24

**Authors:** Kathy E. Raven, Beth Blane, Danielle Leek, Carol Churcher, Paula Kokko-Gonzales, Dhamayanthi Pugazhendhi, Louise Fraser, Jason Betley, Julian Parkhill, Sharon J. Peacock

**Affiliations:** a Department of Medicine, University of Cambridge, Cambridge, United Kingdom; b Illumina, Inc., Cambridge, United Kingdom; c Wellcome Genome Campus, Wellcome Sanger Institute, Cambridge, United Kingdom; d London School of Hygiene & Tropical Medicine, London, United Kingdom; University Hospital Münster

**Keywords:** MRSA, clinical application, whole-genome sequencing

## Abstract

There is growing evidence for the value of bacterial whole-genome sequencing in hospital outbreak investigations. Our aim was to develop methods that support efficient and accurate low-throughput clinical sequencing of methicillin-resistant Staphylococcus aureus (MRSA) isolates. Using a test panel of 25 MRSA isolates previously associated with outbreak investigations, we devised modifications to library preparation that reduced the processing time by 1 hour. We determined the maximum number of isolates that could be sequenced per run using an Illumina MiniSeq platform and a 13-hour (overnight) run time, which equated to 21 MRSA isolates and 3 controls (no template, positive, and negative). Repeatability and reproducibility assays based on this sequencing methodology demonstrated 100% accuracy in assigning species and sequence type (ST) and in detecting *mecA*. Established genetic relatedness between isolates was recapitulated. Quality control (QC) metrics were evaluated over nine sequencing runs. Of the test panel MRSA genomes, 168/173 (97%) passed QC metrics based on the correct species assigned, detection of *mecA* and ST, and depth/coverage metrics. An evaluation of contamination in these 9 runs showed that positive and negative controls and test MRSA sequence files contained <0.14% and <0.48% of fragments that matched another species, respectively. Deliberate contamination experiments confirmed that this was insufficient to impact data interpretation. These methods support reliable and reproducible clinical MRSA sequencing with a turnaround time (from DNA extraction to availability of data files) of 24 hours.

## Abstract (precis)

There is growing evidence for the value of bacterial whole-genome sequencing in hospital outbreak investigations. Our aim was to develop methods that support efficient and accurate low-throughput clinical sequencing of methicillin-resistant Staphylococcus aureus (MRSA) isolates.

## INTRODUCTION

There is growing evidence for the value of bacterial whole-genome sequencing (WGS) in hospital infection control practice and outbreak investigations (1). Numerous retrospective studies have shown that bacterial sequencing provides the discrimination required to distinguish between isolates of the same lineage, overcoming this limitation in previous typing methods (2–7). There is also strong published support for its use in investigating carriage, transmission, and suspected outbreaks in high-risk areas, such as intensive care units (2, 6). Used early, this could lead to action that limits the size of an outbreak (6, 8). Furthermore, sequencing can exclude outbreaks where a cluster of patients positive for the same pathogenic species has arisen by chance (9), saving unnecessary infection control interventions and outbreak investigations.

The benefit gained from using WGS during outbreak detection is likely to be greatest if the technology is embedded within health care institutions and performed with a rapid turnaround time. This has become increasingly feasible through technical advances in sequencing instruments and the availability of commercial kits and liquid-handling robots that simplify DNA extraction and library preparation. The laboratory processing aspects of WGS are now within the capabilities of larger diagnostic laboratories. The technical feasibility of sequencing in real time has been demonstrated previously at a tertiary-care hospital in Germany, but the turnaround time was 4.4 to 5.3 days, with a cost of ∼£170 (10). Reducing this turnaround time to results and the cost of sequencing will be key to implementing sequencing in the clinical setting and having an impact on infection control. In our clinical microbiology laboratory at Addenbrooke’s Hospital in Cambridge, United Kingdom, we are developing the methods and processes to introduce routine WGS of targeted nosocomial pathogens in close to real time to enhance our infection control practice. Here, we describe the development of laboratory processing methodology for low-throughput clinical sequencing of methicillin-resistant Staphylococcus aureus (MRSA) strains.

## MATERIALS AND METHODS

### Test panel isolates.

Twenty-nine bacterial isolates (27 S. aureus and 2 Escherichia coli) were assembled into a test panel for the study (Table 1). The majority of S. aureus (*n* = 25) isolates were MRSA from two evaluations of sequencing at the Cambridge University Hospital NHS Foundation Trust hospital (CUH) (6, 7). Twenty-one MRSA isolates were selected from a 12-month study of MRSA-positive patients (7) to provide representation of the dominant clonal complexes in our setting (clonal complex 22 [CC22], CC30, and CC5), combined with a range of genetic relatedness. A further 4 MRSA isolates (all sequence type 22 [ST22]) were from an outbreak in a special care baby unit (6). Also included were 4 reference isolates, MRSA HO 5096 0412, methicillin-susceptible S. aureus NCTC 6571, E. coli NCTC 12241, and E. coli NCTC 10418. For sequencing, isolates were cultured from frozen stocks onto Columbia blood agar (CBA; Oxoid) and incubated in air at 37°C overnight, and single colonies were picked for DNA extraction and further processing. Table 1 indicates the isolates used in each sequencing run.

**TABLE 1:** Panel of bacterial isolates used in the study

Sample name	ENA/SRA/GenBank accession no.	Control or test isolate	Species	ST	Original study	Transmission cluster	Presence by sequence run no:									
							1	2	3	4	5	6	7	8	9	10
SASCBU35	ERR131801	Test isolate	*Staphylococcus aureus*	22	Harris et al. (6)	Unrelated to cluster 1	x	x		x	x	x	x	x	x	
SASCBU17	ERR072246	Test isolate	*S. aureus*	2371	Harris et al. (6)	1	x	x	x	x	x	x	x	x	x	
SASCBU18	ERR072247	Test isolate	*S. aureus*	2371	Harris et al. (6)	1	x	x	x	x	x	x	x	x	x	
SASCBU25	ERR108054	Test isolate	*S. aureus*	2371	Harris et al. (6)	1	x	x	x	x	x	x	x	x	x	
MPROS0386	ERR212946	Control isolate	*S. aureus*	22	Coll et al. (7)	Unrelated to cluster 2	x	x	x (2)	x	x	x	x	x	x	
MPROS1839	ERR715142	Test isolate	*S. aureus*	22	Coll et al. (7)	2	x	x	x	x	x		x	x	x	x
MPROS2508	ERR715397	Test isolate	*S. aureus*	22	Coll et al. (7)	2	x	x	x	x	x		x	x	x	
MPROS2264	ERR715156	Test isolate	*S. aureus*	22	Coll et al. (7)	2	x	x	x	x	x		x	x	x	
MPROS2239	ERR715240	Test isolate	*S. aureus*	22	Coll et al. (7)	2			x	x	x		x	x	x	
MPROS0292	ERR212846	Test isolate	*S. aureus*	22	Coll et al. (7)	2	x	x		x	x		x	x	x	
MPROS2066	ERR702160	Test isolate	*S. aureus*	30	Coll et al. (7)	3	x	x	x	x	x		x	x	x	
MPROS1560	ERR737278	Test isolate	*S. aureus*	30	Coll et al. (7)	3			x		x		x	x	x	
MPROS0947	ERR714803	Test isolate	*S. aureus*	30	Coll et al. (7)	3	x	x		x (2)	x		x	x	x	
MPROS2402	ERR715316	Test isolate	*S. aureus*	30	Coll et al. (7)	Unrelated to cluster 3	x	x	x		x		x	x	x	
MPROS0541	ERR702114	Test isolate	*S. aureus*	5	Coll et al. (7)	4	x		x	x	x		x	x	x	
MPROS1125	ERR737419	Test isolate	*S. aureus*	5	Coll et al. (7)	4	x		x	x	x		x	x	x	
MPROS0046	ERR212783	Test isolate	*S. aureus*	5	Coll et al. (7)	4			x	x	x		x	x	x	
MPROS0238	ERR204190	Test isolate	*S. aureus*	5	Coll et al. (7)	4			x							
MPROS2412	ERR715326	Test isolate	*S. aureus*	5	Coll et al. (7)	4	x		x							
MPROS0158	ERR211966	Test isolate	*S. aureus*	5	Coll et al. (7)	Unrelated to cluster 4	x		x (2)							
MPROS0688	ERR701921	Test isolate	*S. aureus*	22	Coll et al. (7)	5				x	x		x	x	x	
MPROS2335	ERR736981	Test isolate	*S. aureus*	22	Coll et al. (7)	5				x	x		x	x	x	
MPROS0659	ERR701905	Test isolate	*S. aureus*	22	Coll et al. (7)	6				x	x		x	x	x	
MPROS2044	ERR702173	Test isolate	*S. aureus*	22	Coll et al. (7)	6				x	x		x	x	x	
MPROS1689	ERR737479	Test isolate	*S. aureus*	22	Coll et al. (7)	Not applicable	x	x	x	x						
H050960412	HE681097	Test isolate	*S. aureus*	22	Reference strain	Not applicable	x	x	x	x	x	x	x	x	x	
NCTC 6571	ERR1100774	Test isolate	*S. aureus*	30	Reference strain	Not applicable	x	x								
NCTC 12241	ERR718772	Control isolate	*Escherichia coli*	Not applicable	Reference strain	Not applicable			x (2)	x	x	x	x	x	x	x
NCTC 10418	ERS523599	Control isolate	*E. coli*	Not applicable	Reference strain	Not applicable	x	x								

### Positive and negative controls.

Three controls were included in every sequencing run to monitor the ongoing performance of the entire testing process. These were a no-template control, a positive control (MRSA MPROS0386) that is 115 core genome single-nucleotide polymorphisms (SNPs) different from the MRSA HO 5096 0412 mapping reference, and a negative control (E. coli NCTC 12241). The no-template control contained all assay components except for DNA and was used to verify the lack of contamination across reagents and samples. The positive control was used to control the entire assay process and analytical accuracy. The negative control was used to assess cross-contamination during processing and represented the nontarget DNA sample to verify analytical specificity. In the first two runs, an alternative E. coli control isolate (NCTC 10418) was used, but this had a low match to E. coli in Kraken (∼22%) and was replaced by NCTC 12241 (>50% match). Fresh stocks of molecular-grade water and phosphate-buffered-saline were opened each week. Other “reuse” reagents were checked for bacterial contamination weekly by subculturing using a 1-μl loop onto CBA and incubating overnight in air at 37°C.

### DNA extraction, library preparation, and sequencing.

DNA was extracted using a DNA mini extraction kit (Qiagen) following the protocol “Isolation of genomic DNA from Gram-positive bacteria” in “Appendix D: protocols for bacteria,” with the following amendments: the incubation with proteinase K was performed at 56°C for 30 min, and in the final elutions, 50 μl distilled water was added with the full 5 min of incubation. DNA was quantified using a Qubit fluorometer. Sequencing libraries were made using the Illumina Nextera DNA flex kit based on the manufacturer’s instructions (11), with several modifications to reduce processing time (see Results). In the first 3 runs, the input DNA to library preparation was normalized to ∼100 ng, but thereafter, we used a range of up to 500 ng DNA. Libraries were sequenced on an Illumina MiniSeq platform with a run time of 13 h (overnight) using the high-output 150-cycle MiniSeq cartridge and the Generate Fastq workflow. Genomes were demultiplexed using the Generate Fastq workflow and the data transferred to an external 1-Tb USB-connected hard drive for further analysis. Ten sequencing runs were performed during this evaluation; the objective of each run is summarized in Table S1 in the supplemental material.

### Sequence data analysis.

Multilocus sequence types (STs) of the MRSA isolates were identified using ARIBA version 2.12.1 (https://github.com/sanger-pathogens/ariba/wiki/MLST-calling-with-ARIBA). Species were determined using Kraken version 1 (https://ccb.jhu.edu/software/kraken/) with the miniKraken database (https://ccb.jhu.edu/software/kraken/dl/minikraken_20171019_8GB.tgz). The presence of *mecA* (GenBank accession number HE681097, positions 2790560 to 2792566) was determined using ARIBA, with a minimum percentage identity of 70% required based on a study by Ito et al. (12), and a minimum of 90% of the gene length covered. All isolates were mapped to the MRSA HO 5096 0412 CC22 reference (accession number HE681097) using SMALT (https://www.sanger.ac.uk/science/tools/smalt-0), with mapping and base calling performed as described previously (13), using the modifications of kmer size of 13 and step size of 6. The depth and percent coverage of the mapping reference were determined using the script available at https://github.com/sanger-pathogens/vr-codebase/blob/master/modules/VertRes/Pipelines/Mapping.pm.

### Sequence metrics for controls.

Controls were required to pass the following quality metrics. The MRSA positive control must be best matched to S. aureus using Kraken, assigned to ST22, with *mecA* detected, a minimum mean sequence depth of 20×, and minimum 80% coverage of the mapping MRSA reference genome (HO 5096 0412). The E. coli negative control must have the highest species match to E. coli in Kraken, with *mecA* not detected and no S. aureus ST assigned. The no-template control must have fewer than 95,000 fragments in Kraken matching any bacterial species. MRSA isolates from the test panel were required to pass the following metrics: highest match to S. aureus using Kraken, assigned to the correct ST, *mecA* detected, minimum sequence depth of 20×, and minimum 80% coverage of the mapping MRSA reference genome (HO 5096 0412).

### Optimizing the number of isolates per sequencing run.

We estimated that the maximum number of MRSA isolates in a single sequencing run was 24, based on an expected total data output of 3.3 to 3.8 Gb, an average MRSA genome size of 2.8 Mb (https://www.ncbi.nlm.nih.gov/genome/?term=Staphylococcus%20aureus[Organism]&cmd=DetailsSearch), and a target of ∼50× coverage (24 isolates would provide ∼49× coverage). We estimated that 21 test MRSA isolates and three controls (E. coli, MRSA, and no template) could be included per sequence run. This was evaluated by performing sequencing runs that contained either 14, 18, or 21 test MRSA isolates from the study panel plus the 3 controls. One MRSA isolate from the 21-test-isolate run failed to produce sufficient DNA during extraction, and the E. coli control was included twice.

### Repeatability and reproducibility.

Repeatability was evaluated by sequencing six MRSA isolates (HO 5096 0412, MPROS0386, SASCBU17, SASCBU18, SASCBU25, and SASCBU35) in triplicate in a single sequencing run. For each isolate, frozen stock was subcultured onto CBA and incubated in air at 37°C overnight, and three separate colonies were taken forward for individual DNA extraction, library preparation, and sequencing. Reproducibility was evaluated by sequencing 21 MRSA isolates from the test panel in three independent runs. Each isolate was subcultured onto CBA and incubated in air at 37°C overnight, after which three individual colonies were taken forward for DNA extraction, library preparation, and sequencing, one for each sequence run. The entire process was performed by different laboratory staff on three different days. The resulting fastq files were analyzed as described above. Isolates that failed QC metrics were excluded from further analysis (3/18 and 1/63 test isolates failed the repeatability and reproducibility assays, respectively, based on low depth/coverage).

The definition of a correct result was based on species identification, ST assignment, detection of *mecA*, and identification of genetic relatedness based on the detection of SNPs in the core genome compared to the original sequence and the within-run or between run-replicates. Genetic relatedness was determined based on mapping to clonal complex (CC)-specific references, excluding positions denoted as “N” because of failure to call a base. Each repeat and the original sequence data were mapped to a CC-specific mapping reference using SMALT (MRSA HO 5096 0412 [CC22] for ST22 and ST2371, MRSA252 [CC30, GenBank accession no. BX571856] for ST30, and N315 [CC5, GenBank accession no. BA000033] for ST5). Mobile genetic elements were removed using the files available at https://figshare.com/authors/Francesc_Coll/5727779 and the script available at https://github.com/sanger-pathogens/remove_blocks_from_aln. SNPs were identified using the script available at https://github.com/sanger-pathogens/snp-sites. SNPs were identified based on the following parameters: minimum number of reads matching the SNP, 4; minimum number of reads matching the SNP per strand, 2; ratio of SNP base to alternative base, >0.75; variant quality, >50; and mapping quality, >30.

Diagnostic sensitivity and specificity were calculated using the following definitions: true positives are the number of genetically related isolates based on the original data that cluster together based on the test data, false negatives are the number of genetically related isolates based on the original data that do not cluster together in the test data, true negatives are the number of genetically unrelated isolates based on the original data that do not cluster together in the test data, and false positives are the number of genetically distant isolates based on the original data that cluster together based on the test data (14). Clustering was defined based on three SNP classifications, as follows: (i) recent transmission highly likely, with 0 to 10 SNPs different (based on a median within host diversity of 6 SNPs over a year [7] and an estimated mutation rate of 4 SNPs/core genome/year) [15]; (ii) recent transmission likely, with 11 to 25 SNPs; and (iii) recent transmission possible, with 26 to 50 SNPs different (based on the definition of a cluster described by Coll et al. [7]). Isolates with >50 SNPs different were classified as genetically unrelated.

### Analysis of contamination.

The impact on quality metrics from various levels of DNA contamination during clinical MRSA sequencing was evaluated using intentional spiking experiments. One MRSA isolate from the test panel (MPROS1839 [ST22]) and E. coli NCTC 12241 were cultured, and DNA was extracted and quantified as described above. Donor DNA was inoculated into the recipient sample to achieve a final spiked concentration of 0%, 0.1%, 1%, 10%, or 20% (see Results for details of donor and recipient). Contamination with the spike was defined based on the number and proportion of fragments matching to S. aureus or E. coli based on Kraken. The effect of contamination was evaluated using this metric, together with the proportion of the S. aureus CC22 reference covered during mapping, depth of coverage of the mapping reference, and *mecA* and ST detected by ARIBA. Unintentional contamination from internal controls or external sources was evaluated based on the number and proportion of reads matching to other species in Kraken.

### Data availability.

Sequence data generated during this study are available from the European Nucleotide Archive (https://www.ebi.ac.uk/ena) under the accession numbers listed in Table 1.

## RESULTS

Our aim was to develop methods that would support efficient and accurate low-throughput MRSA sequencing in a routine microbiology laboratory in less than 24 h (from DNA extraction to availability of sequence data). The key goals were to maximize the number of isolates sequenced per run, reduce the processing time for DNA preparation, and evaluate quality controls, precision (reproducibility and repeatability), and contamination.

Maximizing the number of isolates per sequencing run was evaluated by performing sequencing runs that contained either 14, 18, or 21 test MRSA isolates from the study panel plus the 3 controls, which were sequenced using the Illumina MiniSeq platform with a run time of 13 h. The median (range) sequence depths for the test MRSA isolates were 92× (33 to 247×), 63× (45 to 77×), and 65× (18 to 107×), respectively, with a minimum of 87% of the genome covered (Table S2). One isolate in the 21-test MRSA run failed the QC metrics based on depth of coverage (17.9×), which on further evaluation could be explained by low input DNA (Table S2). Based on this, we used 21 test isolates plus 3 controls per run during the remainder of the study.

We sought to make modifications to the manufacturer’s protocol for library preparation (Illumina Nextera DNA flex kit) that would reduce processing time while maintaining performance. We proposed that two steps could be changed, as follows: (i) the tagmentation (TAG program) and tagmentation stop (TSB incubation) steps each require 15 min of incubation, which were reduced to 5 min each; (ii) pooling of libraries is recommended after bead cleanup and size selection, but we pooled libraries after PCR and before the bead cleanup and size selection. Two sequencing runs of 21 test panel MRSA isolates plus 3 controls were compared, one of which used the original protocol and the other which made both changes to the protocol. Data were compared for the quantity of DNA added to the library preparation versus the size of the resulting fastq files and depth of coverage as surrogates for the individual DNA quantity outputs from library preparation, which are unavailable with the modified protocol. Detailed results are provided in Table S3. In summary, a comparison of the original and modified protocols showed negligible difference. The median (range) fastq sizes for the original versus modified protocols were 171 Mb (77 to 208 Mb) following 174 to 480 ng DNA input versus 112 Mb (90 to 133 Mb) following 90 to 384 ng DNA input. The median (range) depths of coverage for the original versus modified protocols were 87× (37 to 99×) versus 56× (43 to 70×), respectively. Together, these resulted in a reduction in processing time from 3.5 to 2.5 h for library preparation, taking the combined time for DNA preparation and library preparation to 4.5 h. Subsequent runs used these modifications.

Repeatability was based on concordance of assay results and quality metrics for six MRSA isolates sequenced in triplicate in a single sequencing run. This demonstrated 100% concordance in assigning species and ST and in detecting *mecA*. Four of the six isolates were drawn from a study that investigated a single outbreak on an intensive care unit (6) and were previously identified as having 0 SNPs different (SASCBU17 and SACBU18), 5 SNPs different (SASCBU25), or being unrelated to the outbreak (SASCBU35, >1,500 SNPs different from other isolates). The remaining two isolates were MRSA HO 5096 0412 and the positive MRSA control (MPROS0386). Zero SNPs were identified between the within-run replicates for all isolates, equating to a repeatability per replicate of 100%. Using the original published sequence mapped to the CC22 reference (HO 5096 0412) as the gold standard, all 6 isolates in triplicate had base calls identical to the original sequence (excluding positions denoted as “N” because of failure to call a base), equating to a repeatability per replicate and per base pair of 100%.

Reproducibility was evaluated by sequencing 21 test panel MRSA isolates in three independent runs. This demonstrated 100% accuracy in assigning species and ST and detecting *mecA*. Eighteen of the 21 isolates represented six distinct outbreaks encompassing four different STs (ST22, ST30, ST5, and ST2371) identified during 12 months of genomic surveillance (*n* = 15) (7) or a single outbreak in an intensive care unit (*n* = 3) (6). Of the remainder, 2 isolates were not involved in these outbreaks based on low relatedness, and 1 isolate was the mapping reference MRSA HO 5096 0412. There were 0 SNPs identified between between-run replicates, providing a reproducibility per replicate of 100%. Using the original published sequence when mapped to the CC22 reference as the gold standard, 18 isolates were identical to the original sequence across replicates. The remaining three isolates showed a difference in SNPs compared with the original sequence: MPROS0292 (ST22) had 1 to 2 SNPs different, one of which was reproduced in all three repeats, and the other was reproduced in two repeats, with an N base call in the remaining repeat. MPROS1125 (ST22) had 1 SNP different in one repeat, with an N base call in the same position in the remaining two repeats. MPROS2335 was identical for two replicates, but the third replicate had 10 SNPs. In comparison to the original sequence, this provides an assay accuracy of 92.3% (60/65 repeats), although the true accuracy is likely to be higher, as the majority of SNPs may be genuine based on their presence among repeats.

We next sought to determine the diagnostic sensitivity and specificity for outbreak detection in each of the three reproducibility runs, using the genetic relatedness established previously (6, 7) as the gold standard (Table S4). All test isolate pairs within each run were in the same genetic relatedness category (0 to 10 SNPs, 11 to 25 SNPs, 26 to 50 SNPs, and >50 SNPs) as isolate pairs in the original data. This was reproducible across all three runs and represents a diagnostic sensitivity and specificity for outbreak detection of 100%, which was retained across a range of definitions for genetic relatedness. The majority of isolate pairs were within 1 SNP of the expected SNP difference based on the gold standard. The exceptions were cluster 3 (2 SNPs different between MPROS0046 and MPROS1125 in two runs relating to failure to call a base at one position, and an SNP in a region that was absent across the replicates but present at low coverage in the original sequence) and cluster 4 (MPROS0688 and MPROS2335; 2 SNPs different in two runs due to two positions at which a base failed to be called, and 6 SNPs different in the final run). The sequence of isolate MPROS2335 was genetically identical to that of a second isolate from the same patient sequenced by Coll et al. (7) but is not included here. From this, we suspect within-host diversity of MRSA in this case and sequencing of different colonies of the same lineage.

Quality control metrics were evaluated for the assay controls and MRSA isolates from the test panel over nine sequencing runs (Table S5 provides further details). All three controls in each sequence run passed the required QC metrics. Of 173 S. aureus test panel MRSA isolates sequenced, 168 (97%) passed the QC metrics. The five failures were based on insufficient depth/coverage associated with low input DNA (*n* = 2) or potential loss of DNA during library preparation (*n* = 3). Excluding these 5 failed isolates and the control isolates, S. aureus was the top match in Kraken in all cases (median [range], 85.8% [77.2 to 89.3%]; median [range] coverage depth, 59× [21 to 247×]; median [range] proportion of the reference genome covered, 94.6% [86.3 to 100%]).

We then undertook deliberate contamination experiments to allow us to estimate the impact of various levels of DNA contamination from internal controls or external sources on quality metrics. The details of the donor and recipient DNA, the concentrations of spiked DNA, and our findings are summarized in Table 2. Contamination of the no-template control with increasing concentrations of MRSA DNA did not lead to the control erroneously passing the QC metrics for MRSA until the final spiked concentration reached 10% or greater. This indicates that contamination of the no-template control at 1% (which equated to 96,671 fragments matching S. aureus in Kraken) can be tolerated. Contaminating the positive MRSA control with increasing concentrations of E. coli DNA demonstrated that this could tolerate up to 10% contamination (which equated to 4.04% of fragments matching E. coli in Kraken) before the MRSA QC metrics were not achieved.

**TABLE 2:** Deliberate contamination of controls and MRSA test isolates

Objective	Recipient	Contaminant	Evaluation of impact	Interpretation
Determine the effect of contaminating the no-template control with increasing concn of MRSA DNA	No-template control	Spiked with MRSA MPROS1839 DNA at final concn of 0, 0.01, or 0.1%	No. of fragments matching *S. aureus*, 6, 6, and 20, respectively, for the 3 concn levels; coverage of mapping reference, 36.8–46.7%; avg depth, 0×; did not pass QC metric for MRSA	No-template control can tolerate up to 1% contamination with MRSA DNA
	No-template control	Spiked with MRSA MPROS1839 DNA at final concn of 1%	No. of fragments matching *S. aureus*, 96,671; coverage of mapping reference, 99.3%; avg depth, 7.5×; did not pass QC metric for MRSA	
	No-template control	Spiked with MRSA MPROS1839 DNA at final concn of 10% or 20%	No. of fragments matching *S. aureus*, 363,031 and 623,855, respectively, for the 2 concn levels; coverage of mapping reference, 99.3% (at both concn); avg depth, 28.3× and 48.6×, respectively; both passed QC metrics for MRSA based on depth/coverage, species identification, assignment to ST22, and detection of *mecA*	
Determine the effect of contaminating the MRSA control with increasing concn of *E. coli* DNA	MRSA control	Spiked with serial *E. coli* NCTC 12241 DNA at final concn of 0, 0.01, 0.1, 1, 10, or 20%	MRSA control passed QC metrics at all spikes except 20%, when the proportion of *S. aureus* genome covered fell from 84.6–91.6% (0–10% contamination) to 77.8% (20% contamination); proportions of fragments matching *E. coli* were 0.44, 4.02, and 8.19 at 1%, 10%, and 20%, respectively	Positive control can tolerate up to 10% contamination with *E. coli* DNA

We also evaluated unintentional contamination in nine runs (excluding the deliberate contamination assay). All E. coli and MRSA control sequence data files contained <0.14% of fragments matching another species (Table S6). For the test MRSA sequence files, matches to other staphylococcal species were identified in over half of the samples (109/173; median, 0.05%; range, 0.01 to 0.48% of fragments). Very low-level matches (0.01 to 0.13%) to other species were also identified in specific files (Table S6). All isolates had less than 0.2% of fragments matching to another species, with the exception of a single reference isolate of methicillin-sensitive S. aureus (MSSA) that had a match of up to 0.48% to Staphylococcus nepalensis. Based on the number of fragments in Kraken for the no-template controls and the proportion of fragments in Kraken for the remaining sequences, this demonstrates that, with the exception of the isolate described above, all controls and test isolates had levels of contamination below 1% (0.4% of fragments) across the nine sequencing runs (Table S6).

## DISCUSSION

Our aim was to develop and describe methods for low-throughput clinical sequencing of MRSA using commercial kits and manual methods. Our rationale was that this could support wider uptake in smaller diagnostic laboratories that are not equipped to undertake high-volume sequencing using automated robots. While liquid-handling robots are essential for high-volume sequencing such as that increasingly performed by public health reference laboratories, the majority of routine clinical laboratories have yet to invest in sequencing pipelines with their associated capital and maintenance costs.

An important objective was to enable a 24-h turnaround time from DNA extraction to availability of sequence data. The combined time for DNA preparation and library preparation is 4.5 h, followed by a 13-h (overnight) sequencing run on the Illumina MiniSeq platform. This would support a pipeline of clinical sequencing in which relevant cultures were identified in a routine laboratory and processed, including sequencing, within a day. The methods described here are based on a single colony, which when implemented in routine practice could be obtained from the original diagnostic clinical plate. This turnaround time, in combination with a rapid automated analysis pipeline, would allow infection control to determine whether patients were involved in an outbreak or not the day after a positive culture. This could allow rapid instigation of enhanced infection control procedures when an outbreak is detected to prevent further spread of the outbreak, as well as prevention of infection control actions such as ward closures if a suspected outbreak could be refuted.

We also maximized the number of MRSA isolates per sequencing run to minimize the cost per isolate. Based on 21 clinical isolates per run with three controls, the price per clinical isolate is currently £70 for DNA extraction, library preparation, and sequencing. While individual hospitals are unlikely to frequently reach 21 clinical MRSA isolates suspected to be involved in an outbreak, we suggest a paradigm shift whereby all patients identified as MRSA positive have an isolate sequenced, after which whole-genome sequence data are used to direct infection control actions. This would reduce the turnaround time for action since current outbreak detection relies on multiple time-consuming steps including manually identifying patients that have been in the same ward at the same time. The use of whole-genome sequencing combined with automated analysis would rapidly pinpoint which patients are involved in an outbreak or not, defining the cases on which infection control needs to act and those that require no action. Both turnaround time and cost represent critical measures for translation to clinical use. Alternative sequencing instruments, such as those from Oxford Nanopore Technologies, provide the option for further reductions in sequencing time (16), and over time, the cost and turnaround time of sequencing will undergo further reductions. As costs fall, lower-throughput technologies, such as the Illumina iSeq 100 system, may become viable for routine use in clinical laboratories with smaller sample numbers.

In this study, we described the use and evaluation of assay controls, examined the impact of contamination on data interpretation, and determined the extent to which we inadvertently contaminated the assay. All three controls passed the required QC metrics in every run, together with 97% of test panel MRSA isolates sequenced. High levels of contamination were required before the controls failed QC metrics, and levels of inadvertent contamination were low. Evaluation of precision showed 100% repeatability and reproducibility in assigning species and ST and detecting *mecA*. SNP detection was 100% repeatable, but reproducibility was less than 100% because of the detection of a small number of SNPs that were not present in the original sequence. These can be explained by minor heterogeneity in colonies prepared for independent sequencing, with similar findings reported previously based on sequencing of a range of bacterial species (14). Importantly, the diagnostic sensitivity and specificity for outbreak detection were 100%, indicating that the data generated accurately determined MRSA relatedness, which supports the use of this assay during outbreak investigation. The parameters evaluated in this study were in line with the workflow for validation of whole-genome sequencing in clinical laboratories described previously, and comparable results were obtained (14).

Our findings indicate that the methods evaluated here can provide high-quality data. The single largest impediment to clinical sequencing is a lack of fully automated data interpretation software that has a rapid turnaround time and is suitable for use by nonexperts. This will need to be addressed for routine clinical sequencing to become viable and is currently being investigated by numerous groups and investigators.

## Supplementary Material

Supplemental file 1

## References

[1] Peacock SJ, Parkhill J, Brown NM 2018 Changing the paradigm for hospital outbreak detection by leading with genomic surveillance of nosocomial pathogens. Microbiology 164:1213–1219. doi:10.1099/mic.0.000700. 30052172 PMC7611365

[2] Gorrie CL, Mirc Eta M, Wick RR, Edwards DJ, Thomson NR, Strugnell RA, Pratt NF, Garlick JS, Watson KM, Pilcher DV, McGloughlin SA, Spelman DW, Jenney AWJ, Holt KE 2017 Gastrointestinal carriage is a major reservoir of *Klebsiella pneumoniae* infection in intensive care patients. Clin Infect Dis 65:208–215. doi:10.1093/cid/cix270. 28369261 PMC5850561

[3] Raven KE, Gouliouris T, Brodrick H, Coll F, Brown NM, Reynolds R, Reuter S, Török ME, Parkhill J, Peacock SJ 2017 Complex routes of nosocomial vancomycin-resistant *Enterococcus faecium* transmission revealed by genome sequencing. Clin Infect Dis 64:886–893. doi:10.1093/cid/ciw872. 28362945 PMC5439346

[4] Eyre DW, Cule ML, Wilson DJ, Griffiths D, Vaughan A, O’Connor L, Ip CLC, Golubchik T, Batty EM, Finney JM, Wyllie DH, Didelot X, Piazza P, Bowden R, Dingle KE, Harding RM, Crook DW, Wilcox MH, Tim EA, Walker AS 2014 Diverse sources of *C. difficile* infection identified on whole-genome sequencing. N Engl J Med 369:1195–1205. 10.1056/NEJMoa1216064 PMC3868928 24066741

[5] Walker TM, Kohl TA, Omar SV, Hedge J, Del Ojo Elias C, Bradley P, Iqbal Z, Feuerriegel S, Niehaus KE, Wilson DJ, Clifton DA, Kapatai G, Ip CLC, Bowden R, Drobniewski FA, Allix-Béguec C, Gaudin C, Parkhill J, Diel R, Supply P, Crook DW, Smith EG, Walker AS, Ismail N, Niemann S, Peto TEA, Modernizing Medical Microbiology (MMM) Informatics Group. 2015 Whole-genome sequencing for prediction of *Mycobacterium tuberculosis* drug susceptibility and resistance: a retrospective cohort study. Lancet Infect Dis 15:1193–1202. doi:10.1016/S1473-3099(15)00062-6. 26116186 PMC4579482

[6] Harris SR, Cartwright EJP, Török ME, Holden MTG, Brown NM, Ogilvy-Stuart AL, Ellington MJ, Quail MA, Bentley SD, Parkhill J, Peacock SJ 2013 Whole-genome sequencing for analysis of an outbreak of meticillin-resistant *Staphylococcus aureus*: a descriptive study. Lancet Infect Dis 13:130–136. doi:10.1016/S1473-3099(12)70268-2. 23158674 PMC3556525

[7] Coll F, Harrison EM, Toleman MS, Reuter S, Raven KE, Blane B, Palmer B, Kappeler ARM, Brown NM, Török ME, Parkhill J, Peacock SJ 2017 Longitudinal genomic surveillance of MRSA in the UK reveals transmission patterns in hospitals and the community. Sci Transl Med 9:eaak9745. doi:10.1126/scitranslmed.aak9745. 29070701 PMC5683347

[8] Toleman MS, Reuter S, Coll F, Harrison EM, Peacock SJ 2016 Local persistence of novel MRSA lineage after hospital ward outbreak, Cambridge, UK, 2011–2013. Emerg Infect Dis 22:1658–1659. doi:10.3201/eid2209.151100. 27268128 PMC4994339

[9] Török ME, Harris SR, Cartwright EJP, Raven KE, Brown NM, Allison MED, Greaves D, Quail MA, Limmathurotsakul D, Holden MTG, Parkhill J, Peacock SJ 2014 Zero tolerance for healthcare-associated MRSA bacteraemia: is it realistic? J Antimicrob Chemother 69:2238–2245. doi:10.1093/jac/dku128. 24788657 PMC4100711

[10] Mellman A, Bletz S, Boking T, Kipp F, Becker K, Schultes A, Prior K, Harmsen D 2016 Real-time genome sequencing of resistant bacteria provides precision infection control in an institutional setting. J Clin Microbiol 54:2874–2881. doi:10.1128/JCM.00790-16. 27558178 PMC5121374

[11] Illumina. 2016 Nextera DNA Flex Library Prep reference guide. Document no. 15027987 v01. Illumina, San Diego, CA http://emea.support.illumina.com/content/dam/illumina-support/documents/documentation/chemistry_documentation/samplepreps_nextera/nexteradna/nextera-dna-library-prep-reference-guide-15027987-01.pdf.

[12] Ito T, Hiramatsu K, Tomasz A, de Lencastre H, Perreten V, Holden MTG, Coleman DC, Goering R, Giffard PM, Skov RL, Zhang K, Westh H, O’Brien F, Tenover FC, Oliveira DC, Boyle-Vavra S, Laurent F, Kearns AM, Kreiswirth B, Ko KS, Grundmann H, Sollid JE, John JF, Daum R, Soderquist B, Buist G, International Working Group on the Classification of Staphylococcal Cassette Chromosome Elements (IWG-SCC). 2012 Guidelines for reporting novel *mecA* gene homologues. Antimicrob Agents Chemother 56:4997–4999. doi:10.1128/AAC.01199-12. 22869575 PMC3457410

[13] Klemm EJ, Shakoor S, Page AJ, Qamar FN, Judge K, Saeed DK, Wong VK, Dallman TJ, Nair S, Baker S, Shaheen G, Qureshi S, Yousafzai MT, Saleem MK, Hasan Z, Dougan G, Hasan R 2018 Emergence of an extensively drug-resistant *Salmonella enterica* serovar Typhi clone harboring a promiscuous plasmid encoding resistance to fluoroquinolones and third-generation cephalosporins. mBio 9:e000105-18. doi:10.1128/mBio.00105-18. PMC5821095 29463654

[14] Kozyreva VK, Truong C-L, Greninger AL, Crandall J, Mukhopadhyay R, Chaturvedi V 2017 Validation and implementation of clinical laboratory improvements act-compliant whole-genome sequencing in the public health microbiology laboratory. J Clin Microbiol 55:2502–2520. doi:10.1128/JCM.00361-17. 28592550 PMC5527429

[15] Holden MTG, Hsu L-Y, Kurt K, Weinert LA, Mather AE, Harris SR, Strommenger B, Layer F, Witte W, de Lencastre H, Skov R, Westh H, Zemlickova H, Coombs G, Kearns AM, Hill RLR, Edgeworth J, Gould I, Gant V, Cooke J, Edwards GF, McAdam PR, Templeton KE, McCann A, Zhou Z, Castillo-Ramirez S, Feil EJ, Hudson LO, Enright MC, Balloux F, Aanensen DM, Spratt BG, Fitzgerald JR, Parkhill J, Achtman M, Bentley SD, Nubel U 2013 A genomic portrait of the emergence, evolution, and global spread of a methicillin-resistant *Staphylococcus aureus* pandemic. Genome Res 23:653–664. doi:10.1101/gr.147710.112. 23299977 PMC3613582

[16] Tyler AD, Mataseje L, Urfano CJ, Schmidt L, Antonation KS, Mulvey MR, Corbett CR 2018 Evaluation of Oxford Nanopore’s MinION sequencing device for microbial whole genome sequencing applications. Sci Rep 8:10931. doi:10.1038/s41598-018-29334-5. 30026559 PMC6053456

